# Lymphangiogenesis and angiogenesis during human fetal pancreas development

**DOI:** 10.1186/2045-824X-6-22

**Published:** 2014-11-01

**Authors:** Matthias S Roost, Liesbeth van Iperen, Ana de Melo Bernardo, Christine L Mummery, Françoise Carlotti, Eelco JP de Koning, Susana M Chuva de Sousa Lopes

**Affiliations:** Department of Anatomy and Embryology, Leiden University Medical Center, Einthovenweg 20, 2333 ZC Leiden, The Netherlands; Department of Nephrology, Leiden University Medical Center, Albinusdreef 2, 2300 RC Leiden, The Netherlands; Hubrecht Institute for Developmental Biology and Stem Cell Research, University Medical Center, Uppsalalaan 8, 3584 CT Utrecht, The Netherlands; Department for Reproductive Medicine, Ghent University Hospital, De Pintelaan 185, 9000 Ghent, Belgium

**Keywords:** Angiogenesis, Lymphangiogenesis, Pancreas, Human, Fetal development

## Abstract

**Background:**

The complex endocrine and exocrine functionality of the human pancreas depends on an efficient fluid transport through the blood and the lymphatic vascular systems. The lymphatic vasculature has key roles in the physiology of the pancreas and in regulating the immune response, both important for developing successful transplantation and cell-replacement therapies to treat diabetes. However, little is known about how the lymphatic and blood systems develop in humans. Here, we investigated the establishment of these two vascular systems in human pancreas organogenesis in order to understand neovascularization in the context of emerging regenerative therapies.

**Methods:**

We examined angiogenesis and lymphangiogenesis during human pancreas development between 9 and 22 weeks of gestation (W9-W22) by immunohistochemistry.

**Results:**

As early as W9, the peri-pancreatic mesenchyme was populated by CD31-expressing blood vessels as well as LYVE1- and PDPN-expressing lymphatic vessels. The appearance of smooth muscle cell-coated blood vessels in the intra-pancreatic mesenchyme occurred only several weeks later and from W14.5 onwards the islets of Langerhans also became heavily irrigated by blood vessels. In contrast to blood vessels, LYVE1- and PDPN-expressing lymphatic vessels were restricted to the peri-pancreatic mesenchyme until later in development (W14.5-W17), and some of these invading lymphatic vessels contained smooth muscle cells at W17. Interestingly, between W11-W22, most large caliber lymphatic vessels were lined with a characteristic, discontinuous, collagen type IV-rich basement membrane. Whilst lymphatic vessels did not directly intrude the islets of Langerhans, three-dimensional reconstruction revealed that they were present in the vicinity of islets of Langerhans between W17-W22.

**Conclusion:**

Our data suggest that the blood and lymphatic machinery in the human pancreas is in place to support endocrine function from W17-W22 onwards. Our study provides the first systematic assessment of the progression of lymphangiogenesis during human pancreatic development.

**Electronic supplementary material:**

The online version of this article (doi:10.1186/2045-824X-6-22) contains supplementary material, which is available to authorized users.

## Background

Type 1 diabetes is an autoimmune disease that progressively destroys insulin-producing β-cells [1]. Given the shortage of organ donors, pluripotent stem cells, such as human embryonic stem cells (hESCs) and human induced pluripotent stem cells (hiPSCs), but also adult stem cells from the pancreas may provide a valuable source of insulin-producing β-cells for cell-replacement therapies as well as for studying mechanisms underlying β-cell pathologies [2–4]. To date, protocols to differentiate β-cells from pluripotent stem cells *in vitro* usually generate immature endocrine cells that constitutively secrete insulin, instead of responding to exogenous glucose levels [5–7]. The maturation of these cells into fully functional β-cells is only augmented after transplantation into (immunocompromised) mice [8–10] and likely depends upon a favorable microenvironment for cell maturation and function.

There are only a few studies addressing human pancreas development due to the restricted availability of human embryos [11–20]. Human pancreas development starts between 26 and 35 days post conception with the emergence of dorsal and ventral buds from the foregut epithelium. At 6 weeks of gestation (equivalent to 4 weeks post conception) the two buds fuse and become a single organ formed by stratified epithelium embedded in mesenchyme. The stratified epithelium will give rise to both the exocrine and endocrine compartments of the definitive pancreas [21].

One important physiological regulator of development and normal function of the endocrine cells of the pancreas is the microcirculation through specialized sinusoidal capillaries that irrigate the islets of Langerhans [22–24]. The endothelial cells of these capillaries are highly fenestrated to facilitate the exchange of signals. The dense network ensures that each endocrine cell (glucagon-producing α-cell, insulin-producing β-cell, somatostatin-producing δ-cell, ghrelin-producing ϵ-cell and pancreatic polypeptide-producing PP-cell) is in close proximity to the circulation [25]. It makes up a considerable part of the islets and it is responsible for critical communication via blood signals between the endocrine and exocrine pancreas and also between the different cell types that populate the islets. After transplantation of islets to the pancreas, angiogenesis is key to restoring proper function [8, 25–28].

Another important physiological aspect that regulates the normal function of the pancreas is the presence of a lymphatic system [29]. The main function of the lymphatic system in the pancreas is to drain excess fluid that can contain both leaking endocrine hormones and exocrine-produced digestive enzymes from the interstitial space [29, 30]. Another important function of the lymphatic system, particularly in the context of transplantation and cell-replacement therapies, is the ability to facilitate the immediate transport of immune cells and soluble antigens from the peripheral tissues to the regional lymph nodes for an appropriate immune response [31]. However, understanding the lymphatic vascular network remained poor due to the lack of specific markers capable of distinguishing lymphatic vessels. Two cell surface proteins, namely lymphatic vessel endothelial hyaluronan receptor 1 (LYVE1) and podoplanin (PDPN), have stimulated lymphatic vasculature research as they are expressed specifically by lymphatic endothelial cells, and not blood endothelial cells [32–34].

Together, the lymphatic and blood vascular system control pancreas homeostasis, including the transport of signals, gases, nutrients, hormones, and circulating cells. Both the blood and the lymphatic, vascular systems are highly branched tubular networks, in which vessels are formed by endothelial cells, but there are fundamental differences in terms of function, morphology, and composition [33, 35, 36]. In contrast to the circular blood vascular network, the lymphatic system is blind-ended, consisting of both lymphatic capillaries, pre-collecting and larger collecting lymphatic vessels that are connected to lymph nodes. A profound understanding of the development of the blood and lymphatic vascular system during pancreas organogenesis and the interaction between these systems and human endocrine cells can provide important information on cell maturation and function both *in vitro* and *in vivo*.

In the human pancreas, the presence of CD34-positive blood endothelial cells has been observed from 8 weeks of gestation [13]; PDPN-positive lymphatic vessels have been reported in the pancreas of a single fetus of 18 weeks of gestation [37] and mentioned briefly in a study of the anatomy of the mesocolon transversum at 13 and 16 weeks of gestation [38]. However, the timing of colonization by lymphatic vessels and its relation to the formation of blood vessels in the pancreas is not known. We therefore studied the temporal and spatial progression of lymphangiogenesis and compared this with angiogenesis during human pancreas development between 9 and 22 week of gestation.

## Methods

### Fetal pancreas collection

Fourteen human fetal pancreatic specimens between 9 and 22 weeks of gestation (W9-W12, n = 5; W14-W22, n = 9) were collected from elective abortion tissue obtained by vacuum aspiration. “Weeks of gestation” used in this study is based on the last menstrual period (LMP), to convert to “weeks post conception” one need to subtract two weeks. This study was approved by the Medical Ethics Committee of the Leiden University Medical Center (protocol 08.087). Informed consent was obtained on the basis of the Declaration of Helsinki by the World Medical Association (WMA). All pancreata were fixed in 4% (w/v) paraformaldehyde (MERCK, Darmstadt, Germany) in PBS overnight at 4°C. Fixation was followed by dehydration in ethanol, xylene and paraffin embedding using standard procedures. Embedding was performed using a Shandon Excelsior tissue processor (Thermo Scientific, Altrincham, UK).

### Histology and immunohistochemistry

Paraffin-embedded tissues were sectioned (5 μm) using a RM2255 microtome (Leica Microsystems GmbH, Wetzlar, Germany) and mounted on StarFrost slides (Waldemar Knittel, Braunschweig, Germany). The sections were deparaffinized and rehydrated by standard procedures, namely in xylene and followed by a decreasing series of ethanol ending by rinsing in distilled water. To assess the morphology of the pancreatic sections, a Haematoxylin (MERCK, Darmstadt, Germany) and Eosin (MERCK, Darmstadt, Germany) staining was performed by standard procedures.

For immunohistochemistry, three methods were used for antigen retrieval depending on the primary antibodies: 1) 12 minutes at 97°C in 0.01M sodium citrate buffer (pH 6.0) followed by cooling down; 2) 12 minutes at 97°C in Tris/EDTA buffer (pH 9.0) followed by cooling down; 3) 5-10 minutes 20 μg/ml proteinase K (Promega, Madison, USA) in TE-CaCl_2_ buffer (pH8.0) at room temperature (RT). After antigen retrieval, the sections were blocked with 1% bovine serum albumin, fraction V (BSA, Sigma-Aldrich, St. Louis, USA) in phosphate buffered-saline (PBS) with 0.05% Tween-20 (Promega, Madison, USA) for 1 hour at RT, and incubated with the primary antibodies diluted in the blocking solution overnight at 4°C in a humidified chamber. The primary antibodies used in this study were: Rabbit anti-alpha smooth muscle actin (1:100, ab5694, Abcam, Cambridge, UK), mouse anti-amylase (1:100, sc46657, Santa Cruz Biotechnologies, Dallas, USA), mouse anti-CD31 (1:100, M0823, Dako, Glostrup, Denmark), mouse anti-CD68 (1:1000, M0814, Dako), rabbit anti-CK19 (1:250, ab52625, Abcam, Cambridge, UK), mouse anti-CK19 (ready-to-use, M0888, Dako), rabbit anti-collagen type IV (1:50, AB748, Millipore, Bedford, USA), goat anti-endoglin (1:100, BAF1097, R&D Systems, Minneapolis, USA), rabbit anti-glucagon (1:200, VP-G806, Vector Laboratories Ltd., Peterborough, UK), rabbit anti-insulin (1:100, sc-9168, Santa Cruz Biotechnologies, Dallas, USA), rabbit anti-LYVE1 (1:100, 102-PA50AG, ReliaTech, Braunschweig, Germany) and mouse anti-podoplanin (1:100, ab77854, Abcam, Cambridge, UK). The secondary antibodies were diluted in blocking solution and applied at RT for 1 hour followed by a nuclear counterstaining with 4′,6-diamidino-2-phenylindole (DAPI, Life Technologies, Carlsbad, USA). The secondary antibodies used were: Alexa Fluor 488 donkey anti-rabbit (1:500, A21206, Life Technologies, Carlsbad, USA), Alexa Fluor 594 donkey anti-mouse (1:500, A-21203, Life Technologies, Carlsbad, USA), and Alexa Fluor 594 donkey anti-goat (1:500, A-11058, Life Technologies, Carlsbad, USA). The sections were then mounted using ProLong Gold (Life Technologies, Carlsbad, USA). As antibody specificity controls, primary antibodies were omitted.

### Imaging

The sections stained for Haematoxylin and Eosin were scanned with the Panoramic MIDI digital scanner (3DHISTECH Ltd., Budapest, Hungary). Selection of the desired areas and adjustments were performed with the Panoramic Viewer (3DHISTECH Ltd., Budapest, Hungary). The immunofluorescence images were acquired with a Leica DM5500 fluorescence upright microscope (Leica, Mannheim, Germany) equipped with a Cool Snap HQ2 CCD camera (Photometrics, Tucson, Arizona, USA) or a Leica TCS SP8 upright microscope (Leica, Mannheim, Germany) operated with the Leica Application Suite Advanced Fluorescence software (LAS AF). Brightness and contrast was adjusted either by using ImageJ (NIH, Bethesda, USA) or Photoshop CS6 (Adobe Systems Inc., San Jose, USA).

### 3D-reconstruction

For 3D-reconstruction, serial paraffin transversal sections (10 μm) of fetal pancreatic specimens at W17 and W21 were immunostained for glucagon and podoplanin as described in histology and immunohistochemistry. The sections were digitalized using the Panoramic MIDI digital scanner (3DHISTECH Ltd., Budapest, Hungary) and the reconstruction was conducted with the Amira 4.1 software (Visage Imaging, Berlin, Germany).

## Results

### Peri-pancreatic and intra-pancreatic mesenchyme in the human fetal pancreas

**Figure 1:**
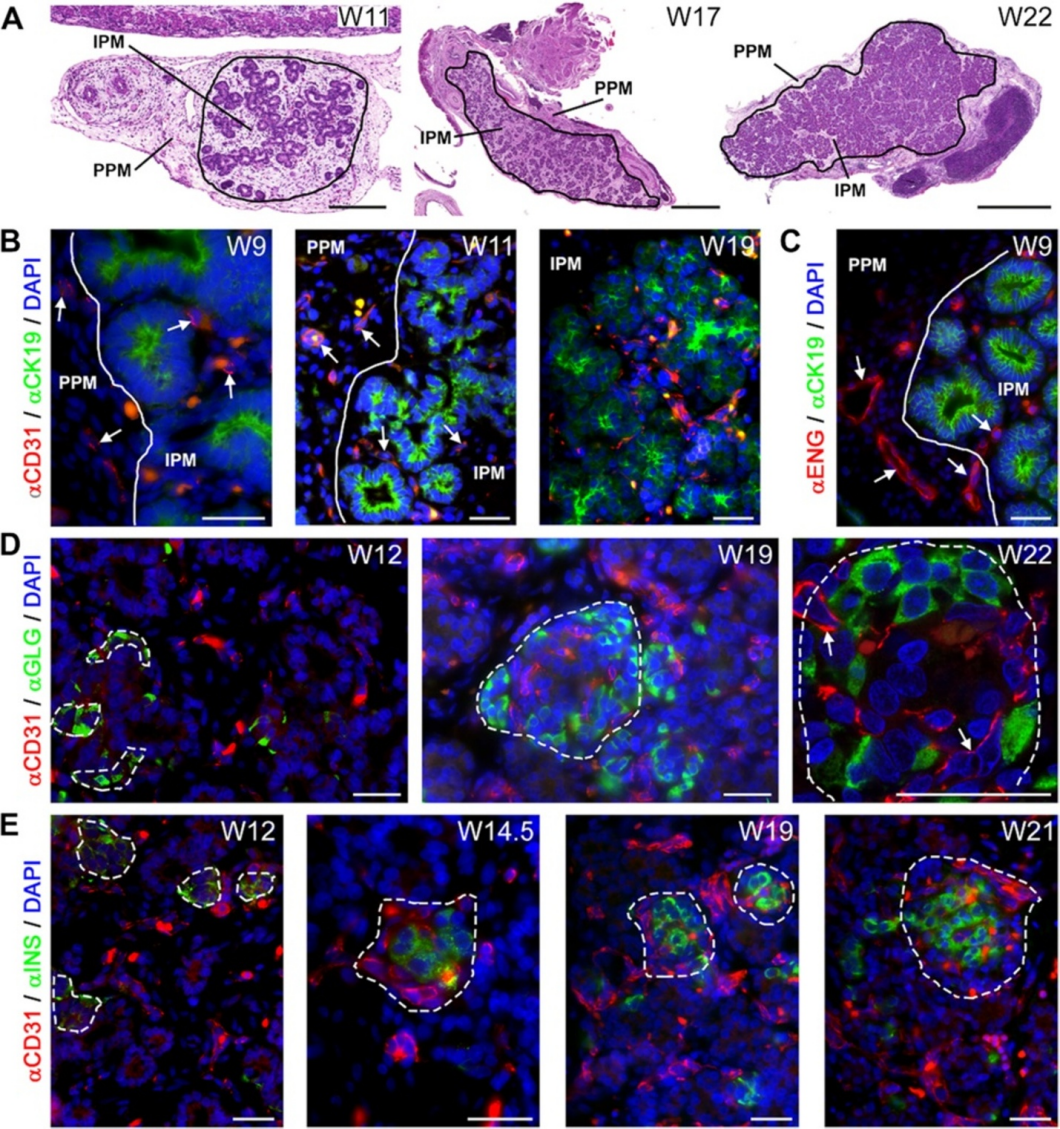
**Angiogenesis during human pancreatic development. (A)** Haematoxylin and eosin (H&E) staining of pancreata at W11, W17 and W22 illustrating the two types of mesenchyme, the peri-pancreatic mesenchyme (PPM) and intra-pancreatic mesenchyme (IPM). The pancreatic epithelium is embedded in the IPM. **(B)** Pancreata at W9, W11 and W19 immunostained for CD31 and CK19. White line in the top panel shows the separation between the PPM and IPM. White arrows point to CD31-positive blood vessels. **(C)** Pancreas at W9 immunostained for endoglin (ENG) and CK19. **(D)** Pancreata at W12, W19 and W22 immunostained for CD31 and glucagon (GLG, α-cells). Dashed line shows the islet of Langerhans. White arrows point to capillaries. **(E)** Pancreata at W12, W14.5, W19 and W21 immunostained for CD31 and insulin (INS, β-cells). Note that autofluorescent red blood cells, as yellow/orange dots, are present in in all images. Scale bars: (A, left panel) 200 μm, (A, middle and right panel) 1 mm, (B-E) 30 μm.

In this study, we refer to two types of mesenchyme in the pancreas (Figure 1A): The peri-pancreatic mesenchyme (PPM) and the intra-pancreatic mesenchyme (IPM). The PPM is the thin layer of connective tissue surrounding the pancreas, whereas the IPM is the connective tissue in which the endocrine and exocrine cells are embedded that will form the (interlobular) septa defining the lobular structure of the adult pancreas.

At W9-W12, the developing pancreas contained a prominent layer of PPM compared to the IPM where the pancreatic epithelium and ducts localize (Figure 1A, left panel). At W17-W22, both types of mesenchyme were less prominent compared to W9-W12, due to the epithelial expansion that leads to endocrine and exocrine differentiation (Figure 1A, middle and right panel).

### Spatial progression of angiogenesis in the pancreatic mesenchymal compartments

To investigate angiogenesis, we used an antibody against CD31 (or PECAM1), an established marker of endothelial cells [34, 39]. From W9-W19, CD31-positive vessels were observed in both the PPM and IPM (Figure 1B), many close to CK19-positive pancreatic epithelial cells. To investigate whether the blood vessels at W9 (the earliest time point analyzed) resulted from angiogenesis or vasculogenesis, we used an antibody against the angiogenic marker endoglin [40, 41]. Already at W9, the blood vessels in both PPM and IPM were endoglin-positive (Figure 1C), suggesting that angiogenesis is the main mechanism for blood vessel formation in the pancreas during the investigated period.

### Microcirculation in the islets of Langerhans

Next, we investigated the relationship between the development of the endocrine compartment and angiogenesis in the developing pancreas. We detected insulin-positive and glucagon-positive endocrine cells within the pancreatic epithelium as early as W9 (data not shown). At W11, the glucagon-positive endocrine cells resided primarily as single cells within the pancreatic epithelium, whereas the insulin-positive endocrine cells already formed small clusters within the pancreatic epithelium (data not shown). At W12-W22, the endocrine compartment of the pancreas developed to form the islets of Langerhans, with the typical core-mantle morphology, with glucagon-producing α-cells forming the mantle (Figure 1D) and insulin-producing β-cells forming a compact core (Figure 1E) as described [13, 20]. Interestingly, a network of CD31-positive capillaries was visible in proximity of the small glucagon-positive and insulin-positive cell clusters around W12 and penetrating the islets, forming the islet microcirculation, at W14.5-W22 (Figure 1D and E). Our observations suggest that at least by W22 the islets of Langerhans and their microcirculation may form a physiological functional unit (Figure 1D, right panel).

### Association of the blood vasculature with smooth muscle cells in the developing human pancreas

**Figure 2:**
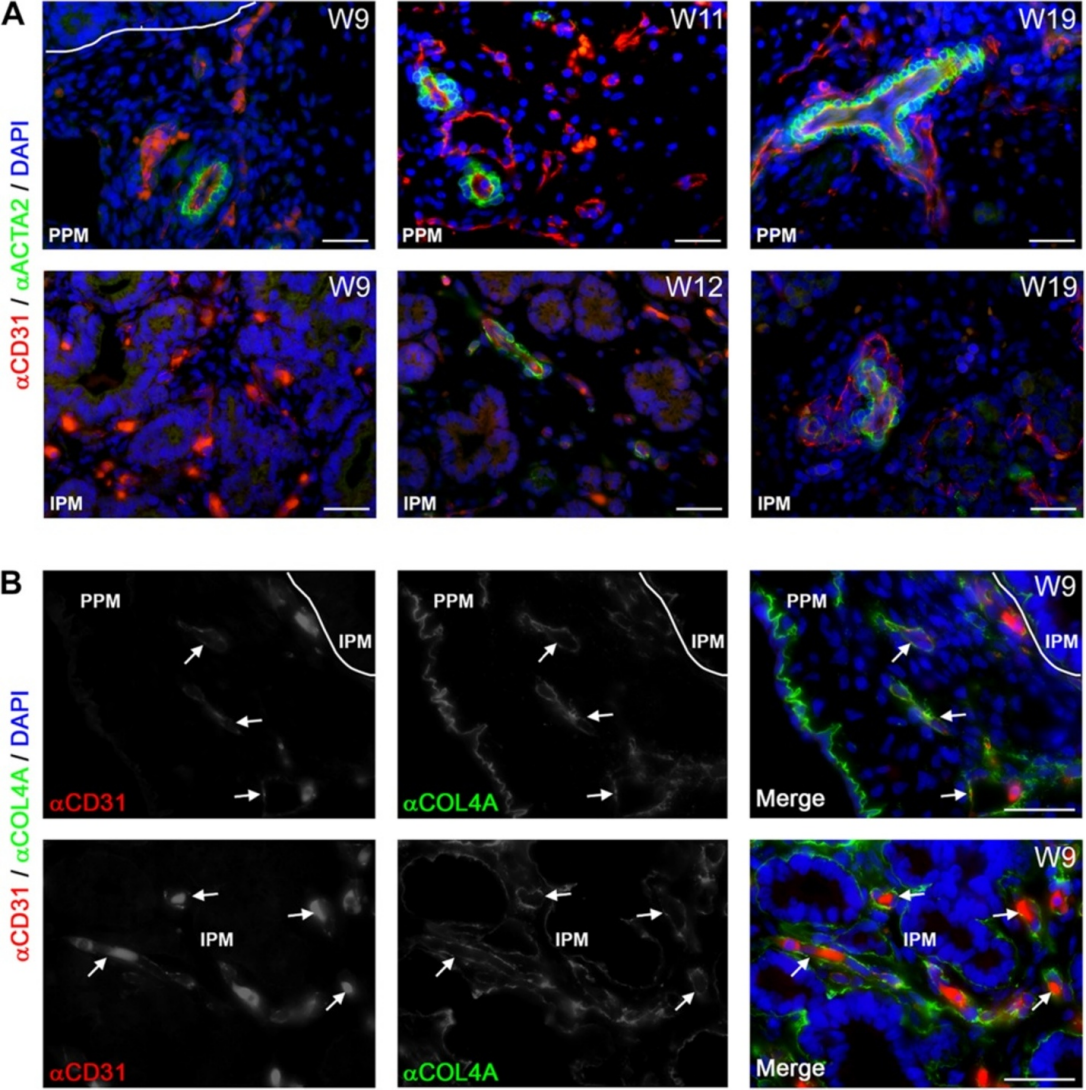
**Blood vessels and their association with smooth muscle cells and basement membrane formation during human pancreatic development. (A)** Pancreata at W9, W11, W12 and W19 immunostained for CD31 and smooth muscle actin (ACTA2). Smooth muscle cell association with blood vessels in the peri-pancreatic mesenchyme (PPM, top panels) and intra-pancreatic mesenchyme (IPM, bottom panels) is shown. **(B)** Pancreas at W9 immunostained for CD31 and collagen type IV (COL4A) illustrating blood vessels in the PPM (top panels) and IPM (bottom panels). White arrows point to CD31-positive blood vessels with a continuous basement membrane. Note that autofluorescent red blood cells, as yellow/orange dots, are present in in all images. Scale bars: 30 μm.

A key step in the maturation of the endothelial tubes to form arteries during vascular development is the attraction of mural cells and their subsequent differentiation to smooth muscle cells through endothelial cell association [42, 43]. The association of smooth muscle cells, expressing alpha smooth muscle actin (ACTA2), with the blood vessels (arteries) was observed as early as W9 (Figure 2A, left panels), but only in the PPM (Figure 2A, left panels). However, from W12 onwards the CD31-positive blood vessels in the IPM also started to show association with ACTA2-positive smooth muscle cells (Figure 2A, middle panels). From W12-W22, the number of CD31-positive blood vessels associated with ACTA2-positive cells, presumably arteries, increased both in the IPM and PPM (Figure 2A, right panels), but some of the large caliber CD31-positive vessels, presumably veins, remained devoid of smooth muscle cells. Large caliber blood vessels (uncoated or coated with ACTA2-positive smooth muscle cells) were never observed in close association with islets of Langerhans. Concluding, the association of smooth muscle cells with blood vessels showed a clear spatial developmental delay of several weeks between the IPM and PPM. By contrast, at W9 blood vessels containing a collagen type IV (COL4A)-rich basement membrane, another key step in the vascular maturation, was already clearly visible in both PPM and IPM (Figure 2B).

### Lymphangiogenesis in the pancreatic mesenchymal compartments

**Figure 3:**
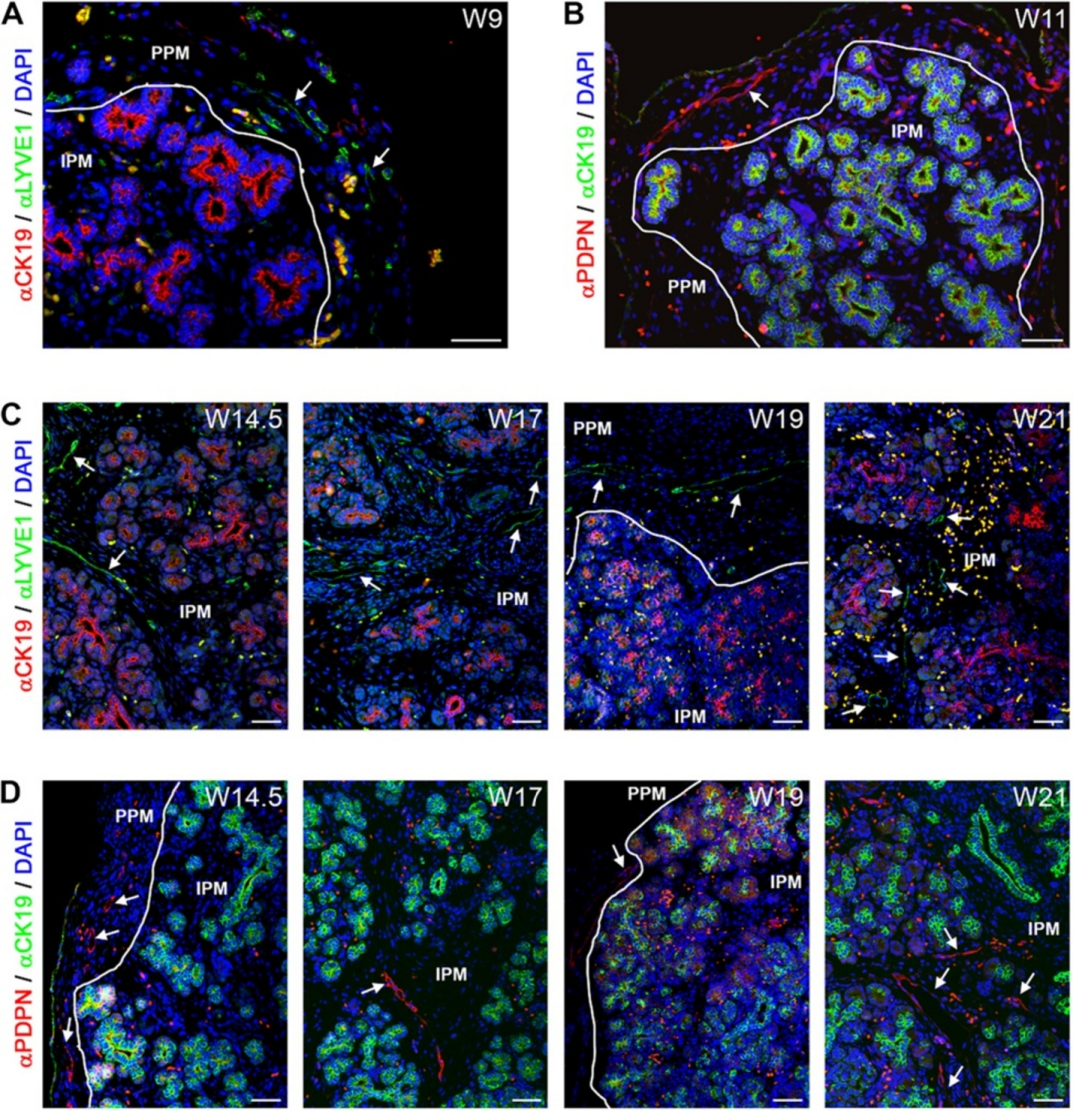
**Lymphangiogenesis during human pancreatic development. (A)** Pancreas at W9 immunostained for CK19 and LYVE1. White arrows point to LYVE1-positive lymphatic vessels. White line shows the separation between the peri-pancreatic mesenchyme (PPM) and intra-pancreatic mesenchyme (IPM). **(B)** Pancreas at W11 immunostained for CK19 and podoplanin (PDPN). White arrows point to PDPN-positive lymphatic vessels. **(C)** Pancreata at W14.5, W17, W19 and W21 immunostained for CK19 and LYVE1. **(D)** Pancreata at W14.5, W17, W19 and W21 immunostained for CK19 and PDPN. Note that autofluorescent red blood cells, as yellow/orange dots, are present in in all images. Scale bars: 50 μm.

From W9 to about W14.5-W17, LYVE1-positive and PDPN-positive small lymphatic vessels were present exclusively in the PPM (Figure 3A and B, white arrows). However, LYVE1, but not PDPN, is also known to be expressed by both CD68-positive and F4/80-positive macrophages [44–46], and those were present as CD68-positive and LYVE1-positive single cells in both the PPM and IPM throughout development (Additional file 1: Figure S1A). By W17, LYVE1-positive and PDPN-positive lymphatic larger caliber vessels were visible in both the PPM and IPM (Figure 3C and D, white arrows), but were rarely or not observed penetrating the intralobular region containing the epithelial-derived tissue (the developing acinar or ductal structures and the islets of Langerhans). The first few amylase-positive acinar cells were observed at W14.5 (Additional file 1: Figure S1B), the stage when lymphatic vessels start to colonize the IPM. However, we did not observe any direct association of lymphatic vessels with amylase-positive cells until W22 either (Additional file 1: Figure S1B).

**Figure 4:**
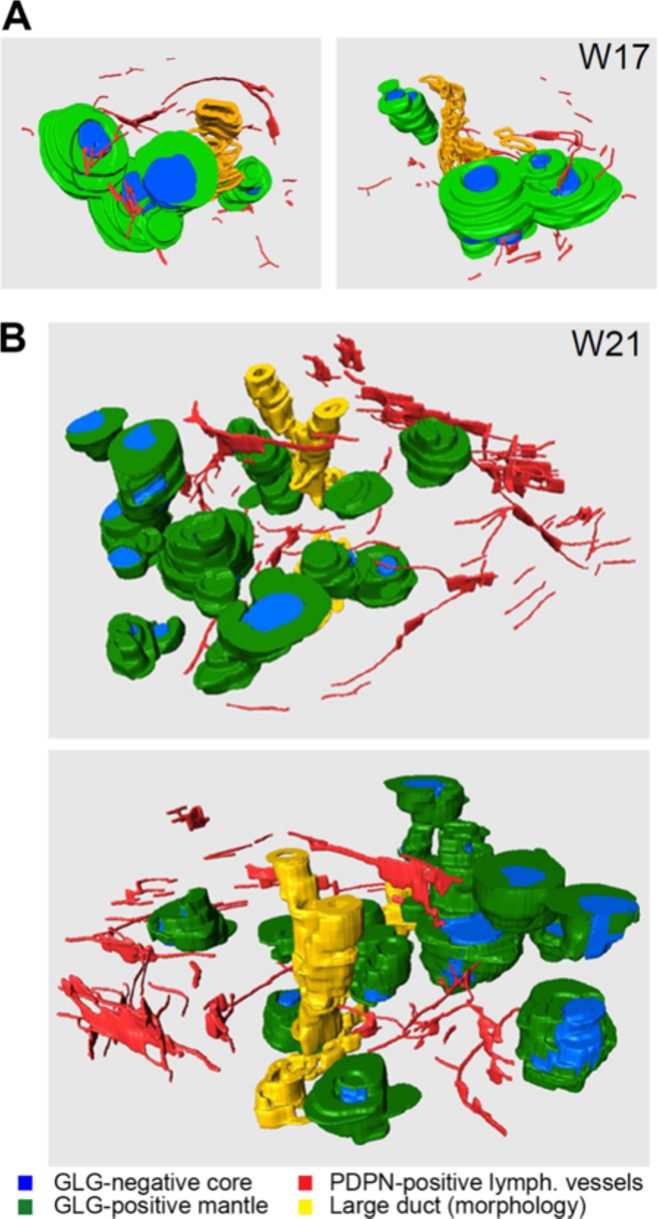
**Spatial arrangement of lymphatic vessels and islets of Langerhans during human pancreatic development. (A, B)** Three-dimensional reconstructions of islets of Langerhans at W17 **(A)** and W21 **(B)**, showing them from two different rotation angles. The mantle of glucagon (GLG)-positive α-cells and the GLG-negative core of the islets of Langerhans are depicted in green and blue, respectively. PDPN-positive lymphatic vessels are colored red and large ducts, identified by their morphology only, are represented in yellow.

Even though there is no direct penetration, from 3D-reconstructions, we observed multiple lymphatic capillaries in close vicinity of islets of Langerhans, at least between W17-W21 (Figure 4A and B; Additional files 2 and 3: Figure S2 and S3). In summary, we observed both lymphatic and blood vessels in the PPM at W9. However, colonization of the IPM by lymphatic vessels (W14.5-W17) was delayed by several weeks compared to the colonization by blood vessels (W9-W11) and was excluded from the islets of Langerhans.

### Association with smooth muscle cells and basement membrane formation in the lymphatic vasculature

**Figure 5:**
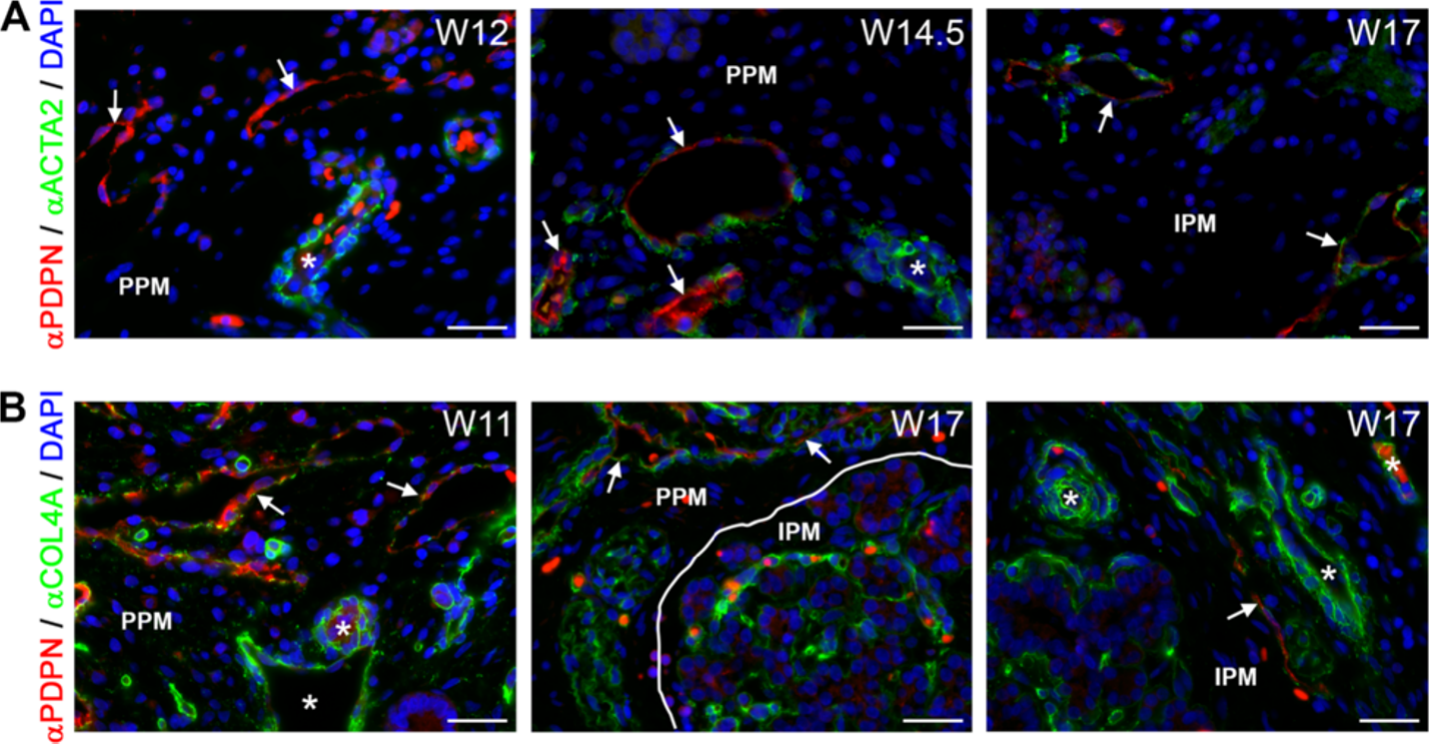
**Association of lymphatic vessels with smooth muscle cells and basement membrane formation during human pancreatic development. (A)** Pancreata at W12, W14.5 and W17 immunostained for podoplanin (PDPN) and smooth muscle actin (ACTA2). PDPN-positive lymphatic vessels (white arrows) showed no association with smooth muscle cells up to W12 in contrast to arteries (white asterisks). From W14.5 onwards, PDPN-positive lymphatic vessels (white arrow) were sparsely covered with smooth muscle cells. **(B)** Pancreata at W11 and W17 immunostained for PDPN and COL4A. PDPN-positive lymphatic vessels (white arrows) were covered with a discontinuous basement membrane, whereas blood vessels (white asterisks) were covered by a continuous basement membrane. Scale bars: 30 μm.

During maturation and remodeling of the lymphatic network, much like the blood vascular network, ACTA2-positive smooth muscle cells are recruited to coat the pre-collecting lymphatics rather sparsely and the collecting lymphatics more densely [47, 48]. In contrast to the association of the blood vascular network with smooth muscle cells observed at W9 in the PPM, the association of ACTA2-positive smooth muscle cells with the PDPN-positive lymphatic vessels was observed only at about W14.5 (Figure 5A), even though both lymph and blood vessels were present in the PPM from W9. In the IPM, the association of lymphatic vessels with smooth muscle cells occurred at about W17, where the largest PDPN-positive lymphatic vessels were only sparsely covered with smooth muscle cells (Figure 5A).

Next, we analyzed the expression of collagen type IV (COL4A), a component of the vascular basement membrane, but also a good indicator of maturation of the lymphatic vessels. COL4A forms a continuous basement membrane in all types of blood vessels and collecting lymphatic vessels, but it forms a discontinuous basement membrane in pre-collecting lymph vessels and does not form any basement membrane in the lymph capillaries [47, 49–52]. By W11, larger-caliber PDPN-positive lymphatic vessels in the PPM showed a discontinuous COL4A-positive basement membrane (Figure 5B, left panel). In the IPM, the invasion by the lymphatic vessels (W14.5-W17) seemed to occur virtually simultaneously with the appearance of a discontinuous COL4A-positive basement membrane (about W17) (Figure 5B, middle and right panels). This discontinuous COL4A-positive basement membrane was maintained at least until W22 in both the PPM and IPM. Together, our data suggest that between W9-W22 there are only lymph capillaries and pre-collecting lymphatic vessels and no collecting lymphatic vessels in the pancreas.

**Figure 6:**
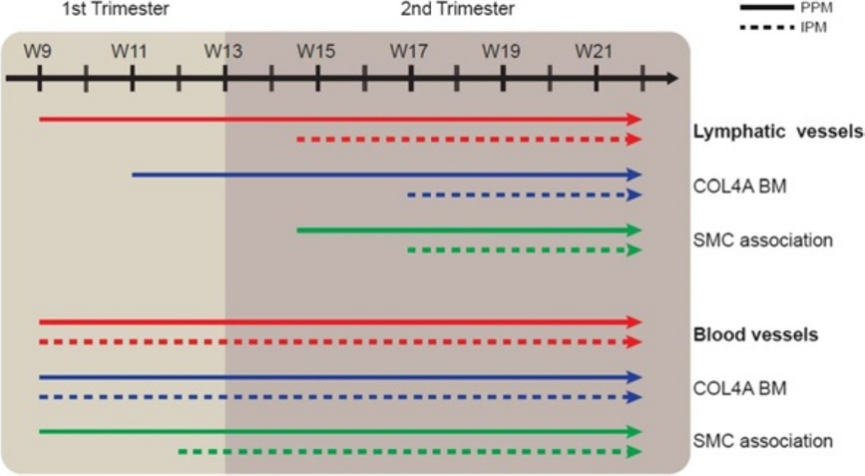
**Model of the progression of lymphangiogenesis and angiogenesis during human pancreatic development.** Cartoon illustrating the progression of lymphangiogenesis (top) and angiogenesis (bottom) showing the timing of invasion, formation of a COL4A-rich basement membrane (BM) and the association with smooth muscle cells (SMCs) in the peri-pancreatic mesenchyme (PPM) and intra-pancreatic mesenchyme (IPM) between W9 and W22.

During pancreas development, lymphangiogenesis occurred rather gradually in the PPM, with invasion at W9, deposition of basement membrane around W11 and coating by smooth muscle cells at W14.5; in the IPM, however, all steps occurred almost simultaneously within 3 weeks, between W14.5-W17 (Figure 6).

## Discussion

Our study showed that in the developing human pancreas, angiogenesis precedes lymphangiogenesis both in the PPM and the IPM (Figure 6). In fact, by W9, mature smooth muscle cell-coated blood vessels were already observed in the PPM, whereas sparsely smooth muscle cell-coated lymph vessels were only observed there by W14.5 (a delay of about 6 weeks). In the IPM, we pinpointed the difference in timing of association with smooth muscle cells to be approximately 5 weeks (W12 for blood vessels and W17 for lymphatics). We conclude that the processes of angiogenesis and lymphangiogenesis followed independent developmental paths both temporally and spatially, which may perhaps be correlated with the development of the endocrine and exocrine compartments between W9-W22. Up to W22, we did not observe any PDPN-positive lymphatic vessels with a continuous layer of smooth muscle cells or a continuous COL4A-positive basement membrane, indicating that the formation of collecting lymphatic vessels may occur only later in development in the pancreas.

We observed proximity between CD31-positive blood vessels and small clusters of glucagon-positive and insulin-positive at (W)12 weeks of gestation (equivalent to 10 weeks post conception) in agreement with Piper and colleagues (2004) that reported proximity between CD34-positive blood vessels at 10.5 weeks post conception [13]. However, the following developmental stage analyzed by Piper and colleagues (2004) was week 14 post conception (equivalent to (W)16 weeks of gestation) when they observed CD34-positive blood vessels penetrating bona fide islets of Langerhans [13]. We now report penetration of the first islets of Langerhans by CD31-positive blood vessels at (W) 14.5 weeks of gestation (equivalent to 12.5 weeks post conception). Furthermore, the immediate establishment of an extensive microvasculature in the islets of Langerhans while these are still being formed confirms the importance of blood vessels as integral part of the islets of Langerhans.

The islets of Langerhans were not directly invaded by lymphatic vessels, but these vessels were clearly present in the IPM in the vicinity of islets by W17. In aggreement, in adult pancreas lymphatic vessels have also been observed not only in the interlobular connective tissue, but also intralobularly where the islets of Langerhans reside [53], suggesting some degree of proximity between lymphatics and islets as in the fetal pancreas. Even though the functionality of fetal islets of Langerhans regarding insulin release in response to glucose by W22 is still a matter of debate [54–57], the vascular machinery to support an insulin-glucose response by the islet of Langerhans seems to be in place.

On the overall morphology of the islets of Langerhans, we report that by W12 the majority of the developing islets of Langerhans exhibited a single core-mantle structure showing a characteristic compact core of insulin-producing β-cells partially surrounded by a thick mantle of glucagon-producing α-cells in agreement with others [13, 20]. However, in contrast to Jeon and colleagues (2009) that observed a predominantly homotypic character in the islets of Langerhans between W18-W21, we observed that the single core-mantle structure was maintained.

From the two lymphatic endothelial cell-specific antibodies used, PDPN is expressed by all lymphatic vessels, whereas LYVE1 is expressed by capillaries and pre-collecting lymphatic vessels, but not by the collecting lymphatic vessels [32, 48, 58], restricting its utility to early stages of lymphangiogenesis. Moreover, we observed that LYVE1, but not PDPN, is also expressed by CD68-positive macrophages as previously described [44–46]. Interestingly, it has been proposed that perhaps these LYVE1-positive macrophages could represent lymphendothelial progenitors [59, 60]. Combining the use of both LYVE1 and PDPN to mark lymphatic vessels provided a robust assessment of lymphangiogenesis in the developing human pancreas.

## Conclusions

We report here the first systematic study investigating the progression of lymphangiogenesis and angiogenesis between W9 and W22 of human pancreas development (Figure 6). We show that both processes have their own dynamics of invasion and maturation, but both seem in place to provide a functional response by W22. Understanding the establishment of the two vascular systems during normal human pancreas development is of great interest to develop better protocols for transplantation of islets of Langerhans, as well as to optimize the molecular niche necessary for the differentiation of pluripotent stem cells to insulin-producing β-cells.

## Electronic supplementary material

Additional file 1: Figure S1: LYVE1-positive macrophages and exocrine differentiation during human pancreas development. **(A)** Pancreata at W9 and W17 immunostained for LYVE1 and CD68, a marker to identify cells of the macrophage lineage (white arrows) in the peri-pancreatic mesenchyme (PPM; top panels) and intra-pancreatic mesenchyme (IPM; bottom panels). **(B)** Pancreata at W12, W14.5, W17 and W19 stained for amylase (AMY) and LYVE1. Scale bars: (A) 30 μm, (B) 50 μm. (TIFF 3 MB)

Additional file 2: Figure S2: Spatial arrangement of lymphatic vessels and islets of Langerhans in a W17 human pancreas. Consecutive sections of a W17 pancreas immunostained for PDPN and glucagon (GLG, α-cells) used for the 3D-reconstruction in Figure 4A. (TIFF 3 MB)

Additional file 3: Figure S3: Spatial arrangement of lymphatic vessels and islets of Langerhans in a W21 human pancreas. Consecutive sections of a W21 pancreas immunostained for PDPN and glucagon (GLG, α-cells) used for the 3D-reconstruction in Figure 4B. (TIFF 2 MB)

## Abbreviations

ACTA2: Alpha smooth muscle actin
BM: Basement membrane
CK19: Cytokeratin 19
COL4A: Collagen type IV
GLG: Glucagon
H&E: Haematoxylin and eosin
INS: Insulin
IPM: Intra-pancreatic mesenchyme
hESCs: Human embryonic stem cells
hiPSCs: Human induced pluripotent stem cells
LYVE1: Lymphatic vessel endothelial hyaluronan receptor 1
PDPN: Podoplanin
PPM: Peri-pancreatic mesenchyme
SMC: Smooth muscle cell
RT: Room temperature.
