# Supplementary figures and images for: Lymphangiogenesis and angiogenesis during human fetal pancreas development
Source: Vasc Cell. 2014 Nov 1;6:22. doi: 10.1186/2045-824X-6-22 (PMC4362646; doi:10.1186/2045-824X-6-22)

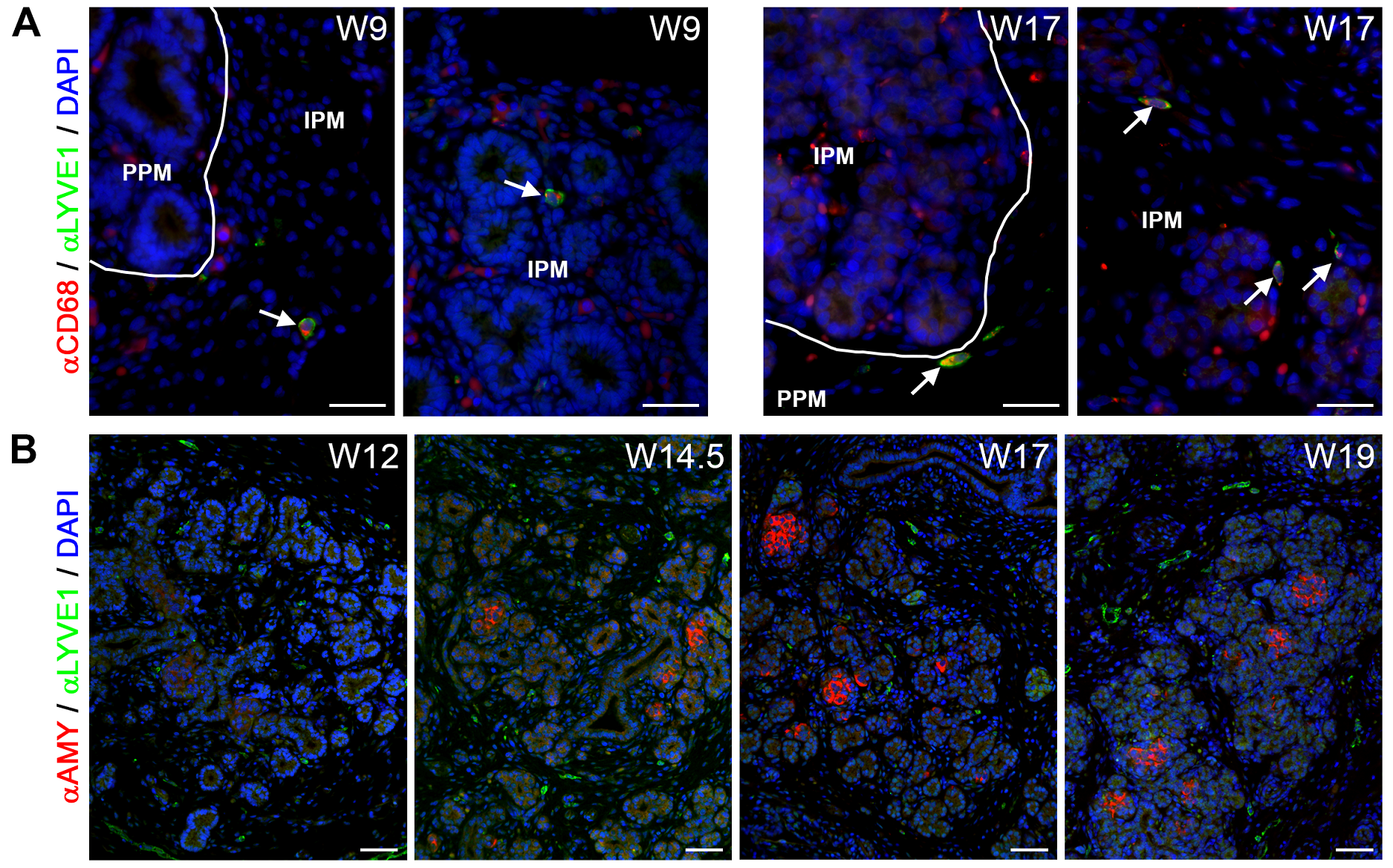

Supplement: Supplementary file 1 — Additional file 1: Figure S1: LYVE1-positive macrophages and exocrine differentiation during human pancreas development. (A) Pancreata at W9 and W17 immunostained for LYVE1 and CD68, a marker to identify cells of the macrophage lineage (white arrows) in the peri-pancreatic mesenchyme (PPM; top panels) and intra-pancreatic mesenchyme (IPM; bottom panels). (B) Pancreata at W12, W14.5, W17 and W19 stained for amylase (AMY) and LYVE1. Scale bars: (A) 30 μm, (B) 50 μm. (TIFF 3 MB) [file 13221_2014_87_MOESM1_ESM.tiff]

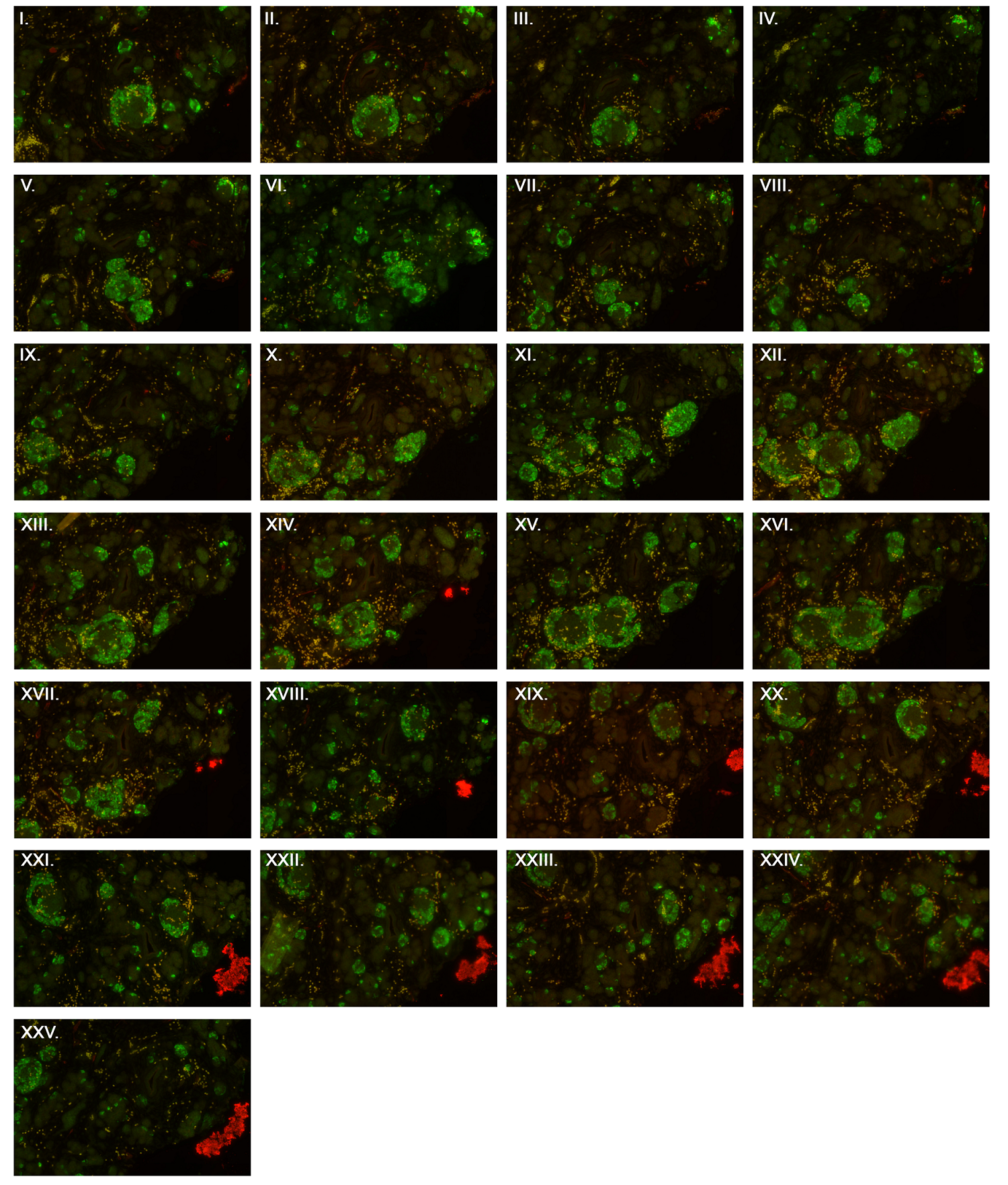

Supplement: Supplementary file 2 — Additional file 2: Figure S2: Spatial arrangement of lymphatic vessels and islets of Langerhans in a W17 human pancreas. Consecutive sections of a W17 pancreas immunostained for PDPN and glucagon (GLG, α-cells) used for the 3D-reconstruction in Figure 4A. (TIFF 3 MB) [file 13221_2014_87_MOESM2_ESM.tiff]

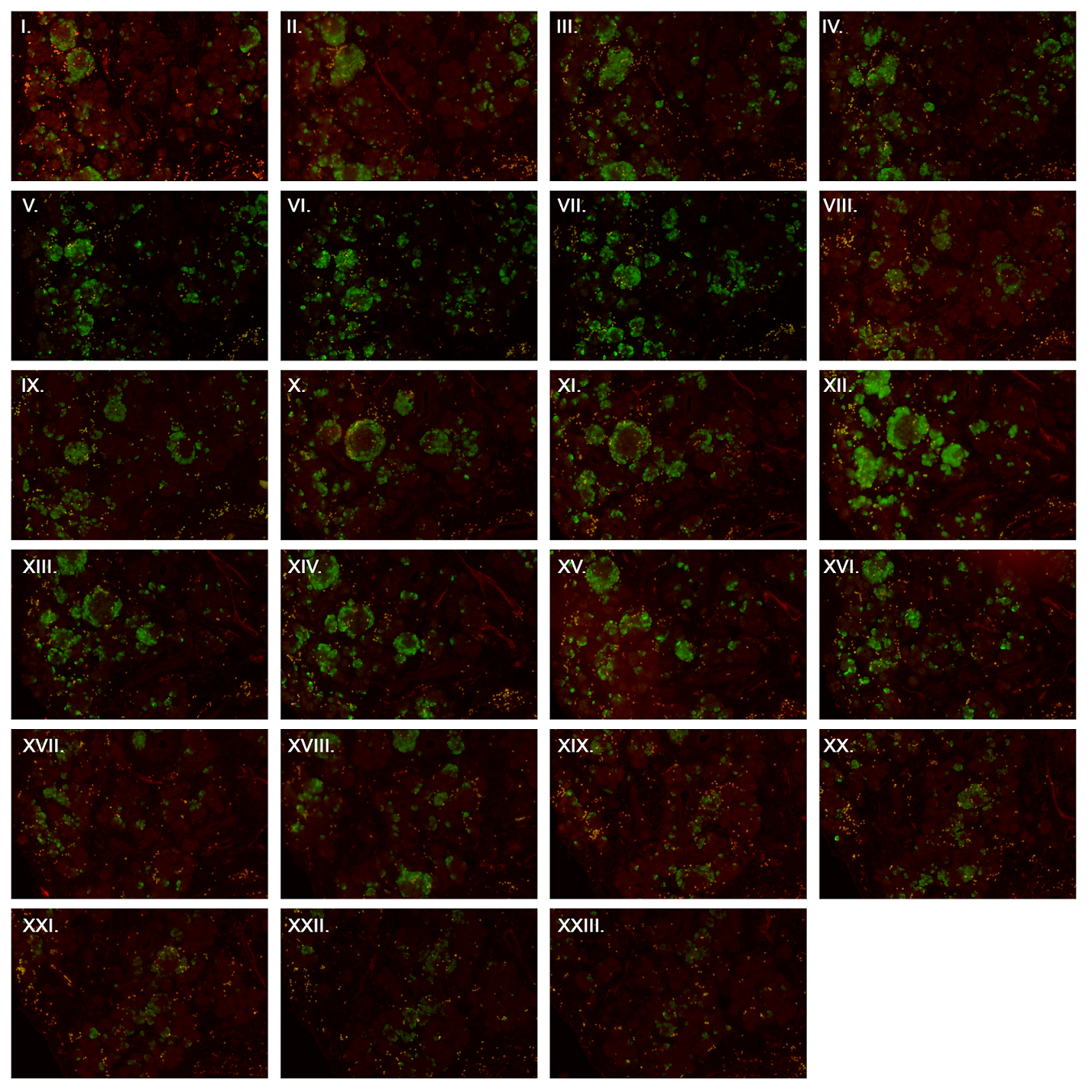

Supplement: Supplementary file 3 — Additional file 3: Figure S3: Spatial arrangement of lymphatic vessels and islets of Langerhans in a W21 human pancreas. Consecutive sections of a W21 pancreas immunostained for PDPN and glucagon (GLG, α-cells) used for the 3D-reconstruction in Figure 4B. (TIFF 2 MB) [file 13221_2014_87_MOESM3_ESM.tiff]
